# Overcoming Lot-to-Lot Variability in Protein Activity Using Epitope-Specific Calibration-Free Concentration Analysis

**DOI:** 10.1021/acs.analchem.3c05607

**Published:** 2024-04-11

**Authors:** Ian B. Harvey, Shannon D. Chilewski, Devyani Bhosale, Sarah E. Tobia, Christopher Gray, Carol Gleason, Jonathan Haulenbeek

**Affiliations:** † Translational Sciences and Diagnostics, Bristol-Myers Squibb, Princeton, New Jersey 08540, United States; ‡ Global Biometrics and Data Sciences, Bristol-Myers Squibb, Princeton, New Jersey 08540, United States

## Abstract

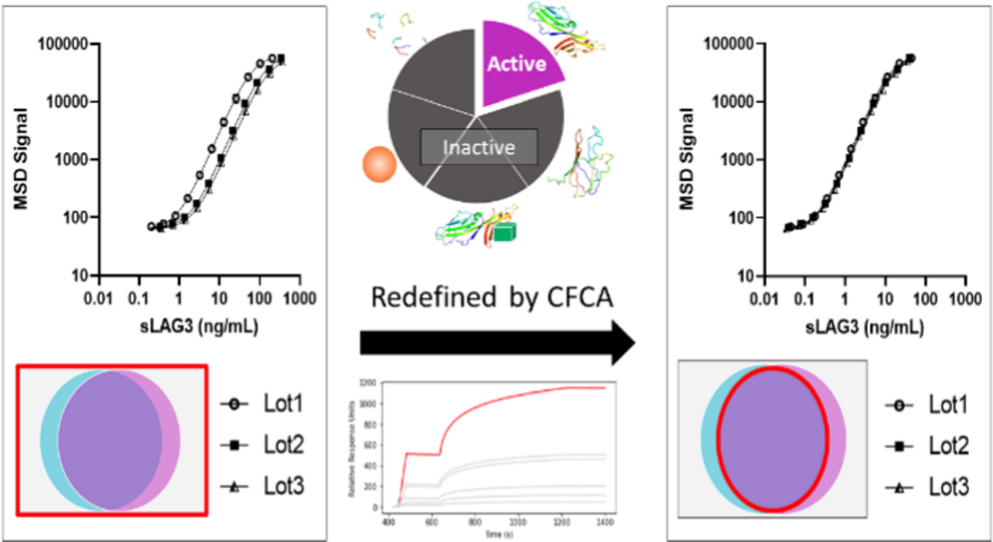

Concentration determination is a fundamental hallmark of protein reagent characterization, providing a means to ensure reproducibility and unify measurements from various assays. However, lot-to-lot differences in protein activity often still occur, leading to uncertainty in the accuracy of downstream measurements. Here, we postulate that those differences are caused by a misrepresentation of the protein concentration as measured by traditional total protein techniques, which can include multiple types of inactive protein species. To overcome this, we developed a standardized method to quantify a protein’s active concentration via calibration-free concentration analysis (CFCA). As a pilot study, we compare the biophysical and immunoassay responses from three batches of recombinant soluble lymphocyte-activation gene 3 (sLAG3), as defined by either their total or active concentrations. Defining the sLAG3 reagents by their assay-specific concentration improved consistency in reported kinetic binding parameters and decreased immunoassay lot-to-lot coefficients of variation (CVs) by over 600% compared to the total protein concentration. These findings suggest that the total concentration of a protein reagent may not be the ideal metric to correlate in-assay signals between lots, and by instead quantifying the concentrations of a reagent’s assay-specific epitopes, CFCA may prove a useful tool in overcoming lot-to-lot variability.

## Introduction

Immunoassays are mainstay platforms for both preclinical and clinical drug discovery/development.^1^ Frustratingly, replacing a recombinant toolkit protein lot can lead to an unacceptable deviation from the original assay response. In regulated bioanalysis, new protein calibrator lots that do not align with the original lot are conventionally either discarded or mathematically bridged by assuming the original lot to be “true” and the new lot to be simply not well characterized.^2−6^ While this method can increase assay precision at each laboratory site, each site may have a distinct original “true” lot of protein calibrator, making comparisons between sites difficult.^7^ Clearly, if clinical teams see the need to systematically “fudge” protein concentration measurements to sufficiently unify data sets, there is a more fundamental issue with the current use of total protein concentration as a correlate of in-assay performance. We therefore sought a concentration determination method that may be able to overcome some of the major drivers of protein lot-to-lot variability.

Lot-to-lot differences in protein potency are likely related to differences in structural integrity and bioactive purity between production lots of a reagent. Reagent proteins are often recombinantly produced, requiring multiple purification steps to obtain acceptably pure material. Even still, protein lots often retain some contaminants and/or partially degraded material.^8^ Conventional protein concentration determination methods such as the bicinchoninic acid (BCA) assay, Bradford assay,^9^ or *A*_280_ measure the total protein in a sample and therefore cannot effectively distinguish between the natively folded protein of interest (POI) and partially/fully denatured protein or contaminants. Considering prior studies that quantify the immunoreactive fraction (IRF) of purified monoclonal antibodies ± radioligands, active concentrations have been reported to range from 35 to 85% of the total protein concentration, with a considerable degree of lot-to-lot variability observed.^10−13^ Thus, lot-to-lot variability in protein purity or activity could translate to variability in assay signals that cannot be accounted for by total protein concentration determination methods.

Calibration-free concentration analysis (CFCA), an alternative method for measuring the concentration of a molecule in solution, was conceptualized for, and is unique to, surface plasmon resonance (SPR) technology.^14−17^ Briefly, this method utilizes saturating levels of a ligand of interest (monoclonal antibody, mAb) on an SPR chip with low concentrations of analyte (recombinant calibrator) so that the system is at least partially mass-transport-limited. Since the physical principles of mass transport in a laminar flow (such as a microfluidic system) have been well described^15,18^ and SPR response is often considered to be linearly related to the mass bound to the surface,^19^ the diffusion and binding of an analyte to a ligand can be kinetically modeled. In this model, if the diffusion coefficient of the analyte, molecular weight of the analyte, and form factor of the SPR chip/system are well defined, the bulk concentration of analyte capable of binding to the ligand can be quantified. This provides a key distinction from total protein concentration quantification methods as only the protein species in an analyte that are capable of binding to the ligand (mAb) on the sensor are quantified by CFCA.

In this study, CFCA was utilized to determine the epitope-specific concentration of recombinantly produced versions of soluble lymphocyte-activation gene 3 (sLAG3), a target of interest in the immuno-oncology space.^20,21^ The capture or detection mAb from a previously validated sLAG3 sandwich immunoassay was used as the ligand; recombinant sLAG3 calibrators were each used as an analyte. Defining the sLAG3 lots by their capture or detection-specific active concentrations yielded more consistent kinetic binding parameters to the respective capture or detection mAbs as compared to using the respective total protein concentrations. While single-epitope active concentrations also decreased the immunoassay variability seen between recombinant lots of sLAG3, sequentially adding a detection mAb injection step after each CFCA cycle allowed for the estimation of the fraction of sLAG3 that could bind detection mAb when sLAG3 was already bound to the capture mAb, the same condition that occurs in a conventional sandwich immunoassay. This additional step further decreased the %CVs between the sLAG3 lots in the immunoassay tested here. Therefore, by quantifying only the concentration of analyte detected in an assay (e.g., biolayer interferometry or immunoassays), CFCA may serve as a potential replacement for total protein assays to overcome multiple confounding issues associated with protein lot variability.

## Experimental Section

### Materials and Methods

The experimental materials, methods, and instrumentation used in this work are detailed in the Supporting Information, Section 1.

### Calibration-Free Concentration Analysis

All CFCA experiments were performed on a Biacore T200 system in 1× PBST running buffer. Where not specified, the CFCA was run by first loading the monoclonal antibody of interest onto a ProteinG-saturated surface, followed by an injection of diluted biomarker at a given flow rate and regeneration of the surface at pH 1.5. In the case of intersection CFCA measurements, a detection mAb injection at 50 μg/mL was also performed in each cycle prior to regeneration. The biomarker dilution series started at 40 or 50 nM and usually did not go below 2–5 nM assuming 100% activity of the reported total concentration. Given that most reagents reported active concentrations greater than 20%, this provides a lower-end cushion to stay within the recommended quantification range of the system (0.5–50 nM). The oligomeric states of glycosylated sLAG3 lots were assigned by SEC-MALS (monomeric). The glycosylated molecular weights for the antibodies and biomarkers used in CFCA fitting were determined using MALDI-TOF analysis. The MALDI-TOF MW was used to predict the diffusion coefficient for each protein^22^ except for NISTmAb which used the CoA-reported hydrodynamic radius. Where necessary, the active concentration molar values were transformed to mass/volume values using the protein sequence MW (unglycosylated), as the Bradford assay specifically measures the quantity of amino acids and is not susceptible to interference from carbohydrates.^9^ CFCA-run validation entailed checking for trace signal intensity and degree of linearity as well as whether the fit had a QC ratio greater than 0.3. Since the CFCA model assumes the system is at least moderately mass-transport-limited, large decreases in mAb loading may negatively impact the experiment. However, this work indicates that ProteinG-based SPR chips could be used for over 1 month, with functional loss being monitored by decreased antibody loading levels. CFCA curve fitting was performed on Biacore T200 Evaluation software version 3.2.1.

## Results and Discussion

### ProteinA- or ProteinG-Conjugated Sensors Provide a Robust Platform to Measure the Active Concentration of Recombinant Calibrators

**Figure 1:**
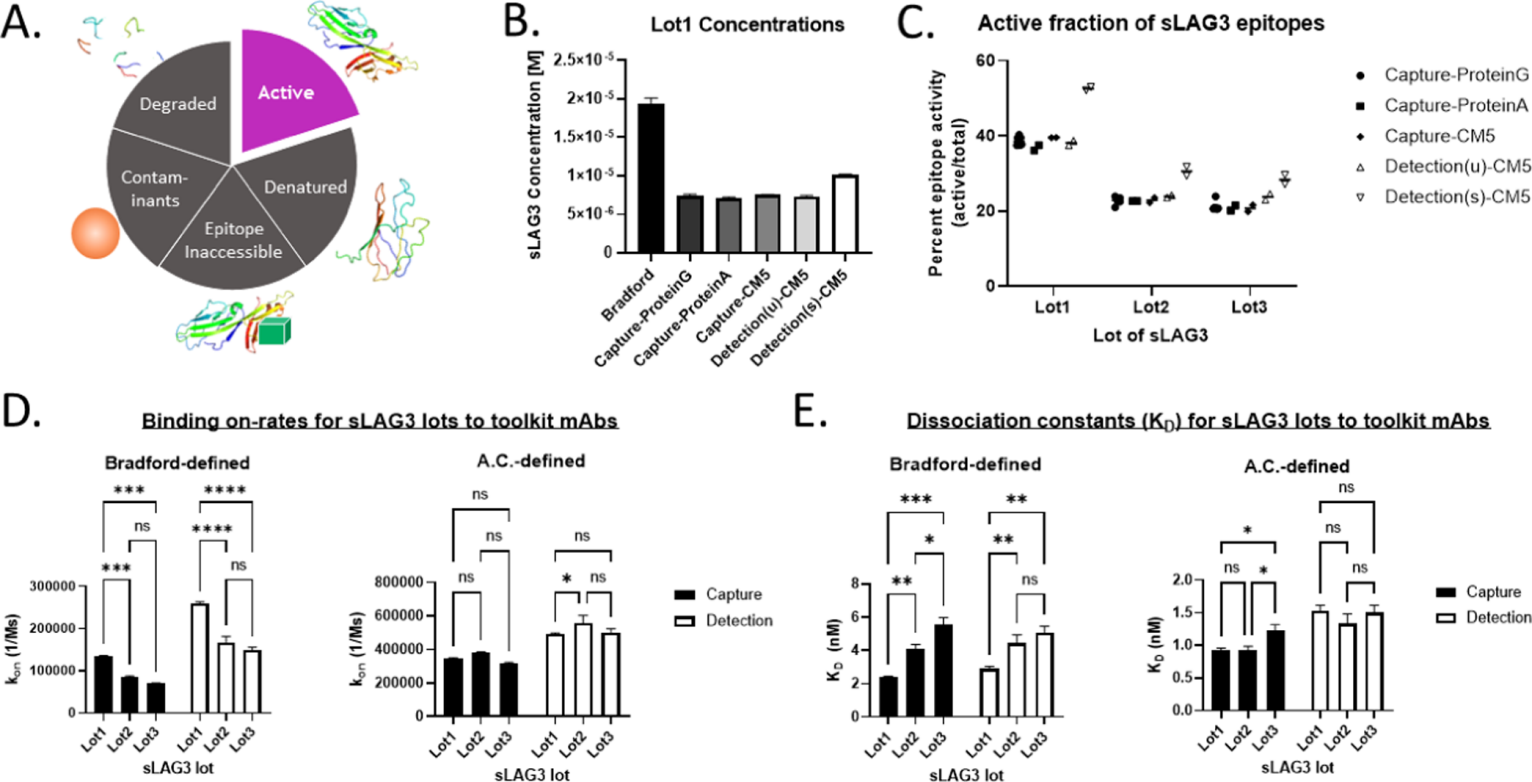
Variability in the percent activity of distinct sLAG3 reagent lots may account for perceived discrepancies in kinetic fits of the respective binding parameters. (A) A pie chart depicting the active and inactive species that may proportionally change between reagent lots, producing lot-to-lot discrepancies in assay responses, using domains of PDB: 7TZG.^24^ (B) The molar active concentrations reported for different epitopes from Lot1 of sLAG3 calibrator (Bradford: *n* = 2, Capture-ProteinG: *n* = 7, Capture-ProteinA: *n* = 2, Capture-CM5: *n* = 2, Detection(u)-CM5: *n* = 2, Detection(s)-CM5: *n* = 2). The Capture-CM5 and Detection(s)-CM5 chips were calibrated with a ProteinG-CM5 chip from the same chip lot, while the Detection(u)-CM5 chip was calibrated with a ProteinA-CM5 chip. Capture mAb was biotinylated, Detection(u) mAb was unconjugated, and Detection(s) mAb was sulfo-tagged. (C) An expansion of the data shown in panel (B) including all three sLAG3 lots, comparing each active concentration value to the respective lot’s Bradford total protein concentration to measure the percent activity of each epitope in each lot. (D) Biolayer interferometry was performed to measure the binding kinetic on-rates of the sLAG3 reagent lots to the capture or detection mAbs. Apparent lot-to-lot discrepancies measured using Bradford-defined concentrations were much less prominent when sLAG3 lots were instead defined by mAb-specific active concentrations (*n* = 2). (E) The respective dissociation constants from the fits in panel (D) similarly demonstrate that using the active concentrations of each sLAG3 reagent leads to increased lot-to-lot agreement compared with using the total concentration.

Initial CFCA experiments were performed using the reference material NISTmAb 8671 to assign form factors specific to each flow cell pair on each sensor type in an effort to standardize downstream CFCA measurements (Results S2.1 and Figure S1). To assess the reusability of these form factors for independent CFCA analyses, the active concentrations of sLAG3 epitopes related to the capture or detection mAb of a previously validated sandwich immunoassay were measured by CFCA. This was achieved by either loading mAbs of interest onto directly calibrated ProteinA/ProteinG sensor chips or, when mAbs were amine-coupled to a CM5 chip, the adjusted form factor from a ProteinA/ProteinG-conjugated CM5 chip in the same lot was used. All active concentration measurements were less than the total protein concentration, suggesting that inactive protein species are present in each reagent lot (Figure 1A–C). Additionally, the active concentrations for the capture mAb epitope measured using a CM5, ProteinA, or ProteinG chip were similar for each lot of sLAG3, emphasizing the utility of independently calibrating each flow cell/sensor (Figure 1B,C).

Unlike the capture mAb, which loaded well to ProteinG (example in Figure S2A), the detection mAb both associated poorly and dissociated relatively quickly from ProteinG (Figure S2B). Therefore, the mass transport limitation was not only lower for the detection mAb bound to ProteinG but also likely changed over the course of the experiment, which may account for the discrepancy between the ProteinG-determined and CM5-determined active concentration for the detection mAb epitope (Figure S2C). Neither ProteinA nor PrismA could provide an acceptable substitute to bind to the detection antibody (Figure S2D), so only the CM5-determined active concentration could be interpreted (Figure 1B,C). While the lots of sLAG3 appear fairly pure by SDS-PAGE (Figure S3A) with calculated purities of 87.2, 85, and 76.7% by SEC-HPLC (Figure S3B), the percent activities of these lots were considerably lower for all epitopes tested either by direct amine-coupling or through ProteinG loading. Low percent activities for recombinant proteins have also been previously reported.^23^ For comparison, we performed CFCA activity assessments of multiple vendor-produced sLAG3 reagents, along with a fourth batch of internally produced sLAG3, produced three months prior to CFCA assessment (Figure S4). Vendor-produced and new internal sLAG3 lots demonstrated higher activities than the one- to three-year-old sLAG3 lots, but none of the reagents had activities in line with the HPLC-measured purities. Interestingly, the percent activity of the detection epitope appeared similar to the capture epitope in each lot when measured using unconjugated detection mAb, but distinct when measured with sulfo-tagged detection mAb (Figures 1C and S4). However, the system precision when reusing CM5 form factors between distinct sensors of a chip lot is currently unclear, so it is difficult to assess whether this discrepancy is due to a calibration issue or whether the sulfotag may interfere with the SPR response.

### Single-Epitope Active Concentrations Improve Lot-to-Lot Consistency in Kinetic Binding Parameter Measurements

The potential value of using CFCA to quantify reagent concentrations stems from the premise that this method exclusively measures protein species that have a functional binding interface of interest. By ignoring protein species that would not produce an assay signal, we hypothesized that CFCA-based concentrations may better harmonize lot-to-lot assay responses if the fraction of inactive species varies (Figure 1C). Therefore, we first sought to assess the variability in measured binding parameters of each sLAG3 lot binding to each toolkit mAb using biolayer interferometry (BLI). Capture and detection mAbs were loaded onto Octet AHC or AMC tips, respectively, dipped into a serial dilution of each sLAG3 reagent lot to monitor association, and finally transferred to buffer alone to monitor dissociation. Each lot’s kinetic traces were then globally fit to a conventional 1:1 binding model, either defining the serial dilution by the respective Bradford or mAb-specific active concentration (Figure 1D,E). Changing the concentration from total to active did not appear to impact the goodness of fit (Figure S5A,C). As initially hypothesized, apparent lot-to-lot variability in binding on-rates or dissociation constants was dramatically decreased when sLAG3 lots were defined by their active concentrations. Additionally, defining lots by these relatively lower concentrations led to increases in reported on-rates and decreases in measured dissociation constants (*K*_D_s) compared to the total concentrations. Since the fitting of an off-rate to kinetic binding data is not influenced by analyte concentration, it is reassuring that we see no appreciable differences between the reported off-rates for the capture or detection mAb, whether we are comparing between sLAG3 lots or between the use of total or active concentrations (Figure S5B).

**Figure 2:**
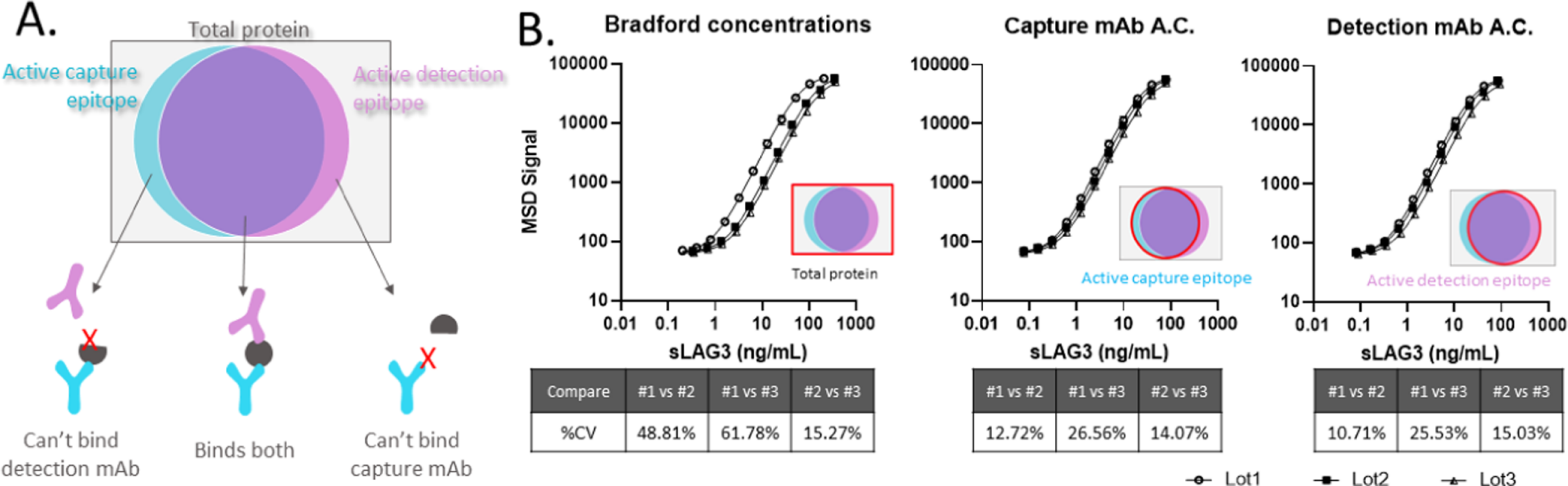
Defining sLAG3 calibrator lots by the capture or detection epitope active concentration moderately unifies immunoassay responses over total protein concentration. (A) Venn diagram representing the overlap of protein species with active capture mAb and/or detection mAb epitopes. (B) MSD titration of three sLAG3 lots, defining each lot by their respective Bradford concentration, capture mAb concentration (Capture-ProteinG), or detection mAb concentration (Detection(s)-CM5). Pairwise %CV comparisons were conducted using data from a prior sLAG3 bridging study, containing responses from 50 patient serum samples. These signals were reinterpolated using the standard curves shown above to have %CVs spanning the clinically relevant range of MSD signals. The overall %CVs for the three lots (100* stdev of all three/mean of all three) were 42.4% for Bradford, 19.0% for capture mAb, and 18.5% for detection mAb.

Considering that sandwich immunoassays rely on two epitopes, each calibrator lot’s assay-specific activity may be better represented by a Venn diagram, encapsulating the active capture epitope concentration and active detection epitope concentration within the total concentration of an sLAG3 lot (Figure 2A). If every protein molecule in a reagent lot were either fully active or fully inactive (no partially folded states), then the capture and detection active concentrations would be equivalent and defining each lot by this concentration would harmonize the immunoassay signals. To assess the use of single-epitope active concentration methods to unify immunoassay calibrator lots, the stock concentrations of each calibrator lot used in an MSD titration experiment were defined as the Bradford, capture epitope, or detection epitope concentration. Both visually and through the coefficients of variation, the active concentration measurements moderately harmonized the sLAG3 lots compared to the Bradford (total) concentration (Figure 2B). However, this unification of lot-to-lot immunoassay signals was incomplete with some coefficients of variation still greater than the generally accepted 15–20% acceptance criteria for bioanalytical experiments.^4^

### Assays Reliant on Multiple Epitopes May Further Benefit from Quantification of Each Reagent’s Intersection Active Concentration

The incomplete lot-to-lot harmonization of sandwich immunoassay responses by relevant single-epitope active concentrations suggests that partially folded sLAG3 species, which contain only one of the two immunoassay-relevant epitopes, may exist in each reagent lot. To exclusively measure the active concentration of sLAG3 species that contain both epitopes (i.e., intersection active concentration), a method was devised to quantify the percent of sLAG3 with an active detection mAb epitope, given the sLAG3 molecule has an active capture mAb epitope.
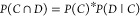
1

In the equation above, *C* refers to the capture mAb epitope being active, *D* refers to the detection mAb epitope being active, ∩ indicates the intersection of two events, and | indicates the conditional that the first event occurs given the second event occurs.

**Figure 3:**
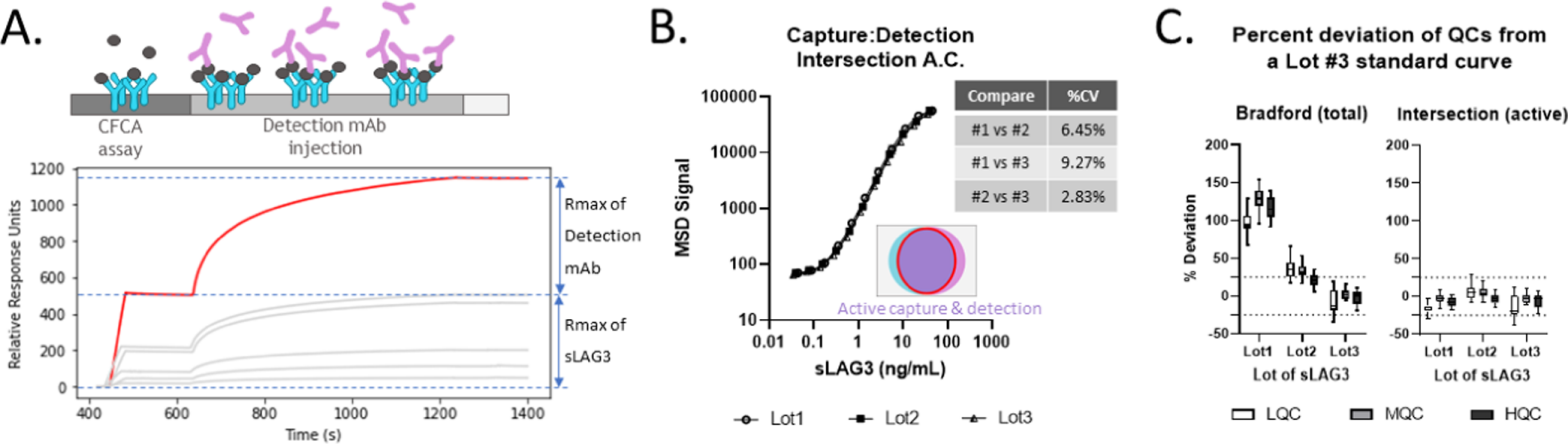
Intersection between the active capture epitope concentration and active detection concentration strongly harmonizes sLAG3 lots by MSD. (A) A CFCA experiment of Lot1 sLAG3 was conducted where an injection of 50 μg/mL detection mAb was appended to each cycle. The highest concentration of sLAG3 loaded onto the surface at 100 μL/min is highlighted in red. The capture mAb was biotinylated, and the detection mAb was unconjugated. (B) The MSD titrations (Figure 2B) were reanalyzed as defined by these intersection active concentrations. An equivalent %CV analysis to Figure 2B found <10% variation between sLAG3 lots using this method. The overall %CV for the three lots (100* stdev of all three/mean of all three) was 6.7%. (C) sLAG3 Lot3 was used to produce a standard curve against which QCs from all three lots were compared. By defining the standard curve and QCs by their Bradford concentrations (left panel), the nonself QC lots are at or above the threshold, 25% deviation from the nominal concentrations. However, when defined by intersection active concentrations (right panel), all three QC lots are within the 25% deviation threshold. This experiment was performed by three different operators over multiple days. QC pairs that did not meet the acceptance criteria of a %CV less than 25% were excluded from analysis.

The conditional probability can be solved experimentally by adding a detection mAb injection at the end of each cycle of a CFCA assay (Figure 3A). The biomarker bound to the capture mAb during the CFCA meets the condition of having an active capture mAb epitope. Given that SPR response is linearly related to mass bound to the chip, a completely active biomarker would be expected to produce a detection mAb secondary injection *R*_max_ that is proportional to the product of the *R*_max_ of the biomarker loaded and the ratio of the two molecular weights.
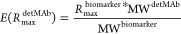
2

After saturating sLAG3 with detection mAb, the experimentally determined maximum detection mAb response can be compared to this ideal max response expected, assuming that each precaptured sLAG3 molecule bound detection mAb 1:1.
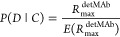
3

Since this detection mAb injection can be described through a bivalent analyte model, the detection mAb concentration must be high to skew toward the mathematically assumed 1:1 detection mAb:sLAG3 binding instead of 1:2. However, at moderate to higher sLAG3 loads, the increased density of sLAG3 on the sensor surface would increase the likelihood that a detection mAb that binds one sLAG3 could sterically reach another. In line with this premise, a moderate negative correlation was observed between the amount of sLAG3 loaded per cycle and the *P*(*D*|*C*) value calculated for that cycle. Therefore, to approximate a reagent’s *P*(*D*|*C*) at 1:1 binding, a linear regression was performed on each set of cycles for each calibrator lot, defining the *Y*-intercept as the *P*(*D*|*C*) value, where avidity-enhancement would be minimized (Figure S6A) and thus 1:1 binding would be maximized. The results of this analysis suggested that nearly half of the sLAG3 calibrator in each lot may not have an accessible detection mAb epitope when the capture mAb is prebound (Figure S6A,B). However, one caveat to this approach is that the *R*_max_ of the detection mAb may not have reached steady state, which would similarly underestimate the *P*(*D*|*C*) of all calibrator lots. While this could be a problem for absolute quantitation, distinct calibrator lots could still be harmonized using this strategy.

Indeed, the sLAG3 calibrator lots strongly converge by MSD when using the intersection active concentrations compared to single-epitope active concentrations or Bradford concentrations (Figure 3B vs 2B). Remarkably, the coefficients of variation drop below 10% for all three calibrator lot comparisons. To demonstrate the utility of this lot harmonization in practice, the sLAG3 MSD assay was performed with a Lot3 standard curve and quality control samples (QCs) diluted from each lot. As expected, QCs prepped from the same lot as the standard curve demonstrate minimal deviation in the back-calculated concentrations (Figures 3C and S7 and Table S1). However, using Bradford-based concentrations, Lot2 QCs were just above the percent deviation threshold of 25% and Lot1 QCs were considerably over this threshold. When these same calibrator reagents were defined by their intersection active concentrations, all three QC lots had percent deviations from the nominal concentrations of less than 25%. Given that the intersection active concentration is an intrinsic quality of each calibrator lot (i.e., does not require comparison to another lot), the unification of interlot MSD signals suggests that adoption of CFCA to define calibrator concentrations may decrease the need to reject new lots of reagents or to conduct bridging/titration studies to equivocate lots.

## Conclusions

Assays that measure binding affinities, as defined by mass action, assume that the defined reagent concentrations are exclusively referencing active/bindable protein species.^25−27^ However, most biophysical studies use the total protein concentration as a proxy for active binding sites, assuming faultless reagent purity and binding site accessibility. This assumption of perfection is rarely achievable in practice, especially when considering heterogeneous proteins (e.g., glycosylated), where a fraction of the protein may have occluded epitopes but be otherwise physiochemically indistinguishable. Because CFCA uniquely quantifies the bindable protein species in a sample, it may better align with the base assumptions used for calculating interaction binding constants than other methods. Prior studies have used CFCA to define protein concentrations for affinity analyses, but the benefits of CFCA over total protein concentration determination were perhaps difficult to discern with a single lot of reagent.^28,29^ In this study, the enhanced lot-to-lot consistency of measured on-rates/*K*_D_s observed using CFCA is likely a result of ignoring impurities/denatured protein species that are incapable of binding and therefore should not be considered when fitting mass action equations.

Given that assay-specific active concentrations ignore protein species that do not bind to the interaction partner or produce a signal in the immunoassay, assay responses fit with the active concentration are likely to be more in line with endogenous protein. Whether recombinant or endogenous, a sample produces a signal only if the assay-relevant epitopes are active. This would particularly be the case if the calibrator and endogenous proteins had similar affinities for the capture/detection mAbs. This raises the point that selection of the most biologically relevant mAb epitopes is paramount when designing an immunoassay because a “total” protein assay may not actually be able to produce a signal for all species of a given endogenous biomarker. Another noteworthy effect of ignoring assay-inactive species when defining protein concentrations would be that the calculated binding affinity decreases, and the sensitivity of the respective immunoassay is seemingly enhanced without modifying the assay itself. In the case of sLAG3, the intersection active concentration accounted for only 10–25% of the total protein concentration, meaning that the MSD assay would have received a 10× to 4× sensitivity increase depending on which calibrator had been originally used in the assay.

While functional assay bridging has been robustly employed to overcome lot-to-lot variability, it requires one lot (often the original) to be considered a gold standard, forcing calibrator agreement by fudging the new lot’s defined concentration based on in-assay responses.^2−6^ This method often results in relatively impressive lot-to-lot %CV values, utilizing the precise conditions of the LBA being bridged. However, functional assay bridging cannot provide an absolute quantification of calibrator concentration, only one relative to the prior lot. This may become an issue when comparing results across multiple sites/studies or if there is no longer any material left from the initial lot, as swapping which lot is considered the gold standard can impact the defined absolute concentrations.^7^ Conversely, the implementation of CFCA described here uses a stable reference standard (NISTmAb 8671) interacting with ProteinA or ProteinG as a means to standardize downstream CFCA measurements. This is akin to the use of a BSA standard to conduct BCA quantification. By utilizing an external reference to standardize SPR responses, the active concentration of each sLAG3 lot is independently quantified. Therefore, the absolute concentrations of the LBA’s calibration curve are less reliant on the order in which the reagent lots are used when employing CFCA compared to functional bridging. This may enhance the reproducibility of results and facilitate analyses across multiple sites or studies.

Aside from harmonizing lots, active concentration may offer additional unique capabilities that are not as feasible with total protein concentration methods. Because the method measures the concentration of a molecule in a solution by a unique antibody:biomarker interaction, it may prove useful in measuring the active concentration of each component in a cocktail for a multiplexed immunoassay (e.g., MSD, Luminex, Olink, PhenomeX). By just changing the antibody being loaded on the chip, a different biomarker concentration could be reported from the same mixed calibrator cocktail. The one stipulation of this is that nonspecific binding would have to be limited, but it is unclear if blocking reagents like BSA could be robustly employed in SPR-based assays to accomplish this. An additional potential use-case for CFCA would be in calibrator/QC stability testing and monitoring. If a large batch of a calibrator shows a decrease in activity over a long storage period, a CFCA reassessment of that lot could provide an updated active concentration of the stock solution that would ignore the protein that has degraded during storage. Indeed, the sLAG3 calibrator lots used in this study were produced from less than one year to over three years prior to this analysis and harmonized well. Any loss in assay-specific activity over the years of storage at −80 °C was therefore likely to have been accounted for by the CFCA. Total protein concentration methods would be useful in this regard only if the inactivated protein precipitated or could otherwise be easily removed from the sample. Active concentration would not require that and so could provide a facile method to dramatically extend the shelf life of calibrators.

It is clear from this work that the CFCA method has been underutilized, given its potential to positively impact the quality and consistency of biophysical analyses and clinical biomarker immunoassays. To the author’s knowledge, this is the first study to describe a method to standardize CFCA measurements and demonstrate the subsequent utility of CFCA in overcoming lot-to-lot reagent variability across multiple assay platforms. Many methods have been applied to reagent generation and characterization to ensure a certain degree of quality and consistency.^30−33^ But lot-to-lot variability in potency often remains, perhaps due to minor deviations in protocols and/or the natural heterogeneity of the expression systems used to produce protein reagents. While controlling expression, purification, and storage conditions are and will continue to be critical to ensure reagent quality, CFCA provides a complementary means to focus the definition of a protein concentration on only the species that can produce a response in the assay-of-interest. Thus, CFCA may prove to be generally applicable to overcome lot-to-lot variability and harmonize signals from protein reagents used in ligand binding assays.

## Supporting Information Available

Supporting Information and methods; standardization of flow cell/sensor signals for CFCA: theory and practical application using NISTmAb 8671; additional analyses regarding sulfo-tagged mAb CFCA, sLAG3 reagent purity/activity, BLI kinetic fits, effect of sLAG3 load on *P*(*D*|*C*) calculation, and QC percent deviations using Bradford or active concentrations; discussions as to alternative potential methods of measuring the intersection active concentration and areas of improvement to enhance CFCA accuracy (PDF)
