# Supplementary material for: Overcoming Lot-to-Lot Variability in Protein Activity Using Epitope-Specific Calibration-Free Concentration Analysis
Source: Anal Chem. 2024 Apr 11;96(16):6275–81. doi: 10.1021/acs.analchem.3c05607 (PMC11044105; doi:10.1021/acs.analchem.3c05607)
Supplement: Supplementary file 1 — ac3c05607_si_001.pdf [file ac3c05607_si_001.pdf]

Supporting Information

Overcoming lot-to-lot variability in protein activity using epitope specific calibration-free concentration analysis

Ian B Harvey*[1], Shannon D Chilewski[1], Devyani Bhosale[1], Sarah E Tobia[1], Christopher Gray[1], Carol Gleason[2], Jonathan Haulenbeek[1]

[1] Translational Sciences and Diagnostics, Bristol-Myers Squibb, Princeton, NJ, USA

[2] Global Biometrics and Data Sciences, Bristol-Myers Squibb, Princeton, NJ, USA

* Author for correspondence:

Email: ian.harvey@bms.com

1 Table of Contents

1 SUPPLEMENTAL MATERIALS AND METHODS 2

1.1 Materials – Reagents and Chemicals 2
1.2 Antibodies and Proteins 2
1.3 Biolayer Interferometry (BLI) kinetics 2
1.4 sLAG3 Ligand Binding Assay 2

2 SUPPLEMENTAL RESULTS 3

2.1 Flow cell form factors can be calibrated using NISTmAb 3
2.2 Supporting data for Figure 1 5
2.3 Supporting data for Figure 2 7
2.4 Supporting data for Figure 3 8
2.5 Supporting data for Supplemental Discussion 10

3 SUPPLEMENTAL DISCUSSION 12

3.1 Alternative methods to determine the intersection active concentration of a calibrator 12
3.2 Areas for method improvement to enhance measurement accuracy 12

4 SUPPLEMENTAL REFERENCES 15

1 SUPPLEMENTAL MATERIALS AND METHODS

1.1 Materials – Reagents and Chemicals

10X Phosphate buffered saline with 0.5% Tween-20 (PBST) (Rockland antibodies and assays #MB-075-1000) was diluted 10-fold in milliQ water and used as the running buffer for all SPR experiments. Series S Sensor Chip CM5 (Cytiva BR100530), Series S Sensor Chip ProteinG (Cytiva 29179315), Series S Sensor Chip ProteinA (Cytiva 29127556) and Series S Sensor Chip PrismA (Cytiva 29650263) were used in this work. Sodium acetate buffer pH 5.0 (Sartorius #18-1069) and pH 4.0 (ForteBio #18-1068) were used for conjugation of antibodies and Protein G (ThermoScientific #21193) respectively. EDC and NHS from the Amine Coupling Kit (Cytiva BR100050) were used to activate CM5 surfaces, while 1M Ethanolamine pH 8.5 (ForteBio 18-1071) was used to quench following conjugation. Glycine pH 1.5 (22053613-AF) and pH 2.0 (GE Healthcare BR-1003-55) were used to regenerate ProteinG and antibodies, respectively.

10X HBS-EP+ (GE Healthcare BR1006-69) was diluted 10-fold in milliQ water, diluted 9:1 into KPL 10% BSA Diluent Blocking Solution (SeraCare 5140-0008) and used for all BLI experiments. Octet® Anti-Human Fc Capture (AHC) (Sartorius 18-5060) or Octet® AMC Biosensors (Sartorius 18-5090) were used in this work for the capture and detection mAbs, respectively.

MSD Gold 96-well streptavidin SECTOR® plates (Mesoscale Discovery L15SA-1) were used to perform the ligand binding assays. Blocker Casein (Thermo Scientific 37528) was used to block the plate after capture. The sample diluent for sLAG3 is comprised of Diluent 3 (Mesoscale R51BA) and Reagent Additive 1 (R&D Systems DY005). MSD Read Buffer T (4X) with Surfactant (Mesoscale Discovery R92TC-1) was used for reading the signal.

1.2 Antibodies and Proteins

NISTmAb, Humanized IgG1k Monoclonal antibody (NIST 8671, lot 14HB-D-002) was used as a standard for the CFCA measurements. Biotinylated recombinant human anti-sLAG3 monoclonal capture antibody (BMS) as well as the unlabeled and sulfo-tagged recombinant mouse anti-sLAG3 monoclonal detection antibody (BMS) were used for the sandwich immunoassay in concert with each of the recombinant human sLAG3 calibrator lots. The sLAG3 calibrator construct (M1-H449) is a full-length ectodomain expressed from stable-expressing CHO-S cells and purified without the signal peptide (L23-H449) through affinity chromatography of a C-terminal 6-His tag followed by size exclusion chromatography. Lot 1 was purified in December of 2019, Lot 2 was purified in February of 2020, and Lot 3 was purified in July of 2022. Calibrators were stored in buffer at -80°C for long-term storage, sub aliquoted and refrozen once prior to analysis, and each subaliquot was then used within 2 weeks of thawing, stored at 4°C. All CFCA and MSD analyses for this study were conducted from Q1 to Q3 of 2023.

1.3 Biolayer Interferometry (BLI) kinetics

All BLI experiments were conducted on a Sartorius Octet HTX system in 1X HBS-EP + 1% BSA. Octet AHC or AMC tips (for capture mAb or detection mAb, respectively) were first equilibrated offline with 1X HBS-EP + 1% BSA for 10 minutes or more prior to usage. sLAG3 reagents were then serial diluted into 1X HBS-EP + 1% BSA based on their total protein concentrations. Following a 2 minute on system equilibration, toolkit mAbs were loaded onto the tips at 10ug/mL for 10 minutes, followed by a 2-minute baseline. The tips were then assayed with a 10-minute association and dissociation phase in the sLAG3 serial dilutions. Curves were globally fit using a 1:1 binding model in ForteBio Data Analysis Version 12.0.2.3. Lot-to-lot differences in binding parameters were assessed using the 2-way ANOVA multiple comparisons test in GraphPad Prism.

1.4 sLAG3 Ligand Binding Assay

Free sLAG3 was measured using the MSD platform as previously described.[1] Briefly, biotinylated capture mAb was loaded on streptavidin-coated MSD plates. Following incubation at room temperature (RT) for 1 to 2 hours, the plates were washed with PBST and blocked with Blocker Casein for 2 to 2.5 hours at RT. Following plate wash with PBST, the standards and QCs were added to each plate, diluted in MSD Diluent 3/Reagent Additive 1 and incubated at RT for 1 hour. The plates were then washed with PBST and the sulfo-tagged detection antibody was added to the plate and incubated for 1 hour at RT. Following the final plate wash with PBST, MSD Read Buffer T was added to each well and the plates were read on an MSD SECTOR® Imager 6000. Data analysis was performed using GraphPad Prism.

2 SUPPLEMENTAL RESULTS

2.1 Flow cell form factors can be calibrated using NISTmAb

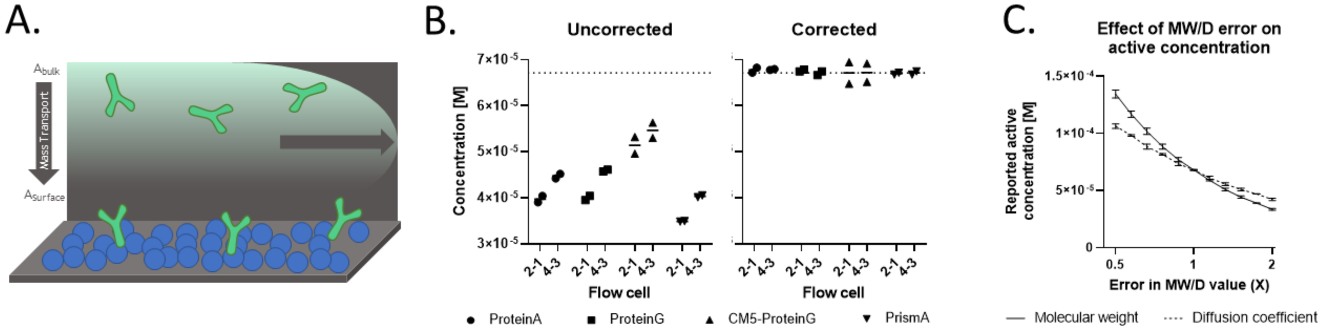

Figure S1: Calibration of Biacore sensor flow-cell pairs using the NISTmAb ProteinG/A interaction unifies the reported active concentrations. (A) A representative CFCA assay where an Fc-binding protein such as ProteinG is conjugated at high density to the surface and an antibody of interest is flowed over the surface, creating a concentration gradient based on its diffusion rate, used to calculate the active concentration. (B) The reported active concentrations of NISTmAb using different sensor types and flow cell pairs on the same Biacore T200 instrument using the default 0.81 form factor, two independent replicates (left panel). Dotted line represents the CoA-reported total concentration for NISTmAb. After applying adjusted form factors to respective chip flow cell pairs, the new reported active concentrations for NISTmAb are in strong agreement (right panel). (C) To see how strongly errors propagate in CFCA, the measured diffusion coefficient or molecular weight was modified (0.5X to 2X artificial error) and the corrected ProteinG 2-1 NISTmAb datasets were reanalyzed. Errors in the molecular weight appeared to have a slightly greater impact on the reported active concentration compared to diffusion coefficient.

A major assumption in CFCA is that analyte binding is at least partially mass transport limited (MTL). This is achieved by saturating the sensor surface with ligand (Figure S1A). However, if there is a marked loss in conjugated ligand activity between cycles (e.g. non-ideal regeneration conditions), later cycles may not have the same degree of MTL as earlier cycles, skewing the resulting active concentration measurement. Previous work has suggested that comparable CFCA results can be attained by capturing the ligand on the chip each cycle prior to analyte injection.[2] To increase robustness and avoid regeneration condition scouting, this study sought to load the antibodies of interest onto ProteinA and/or ProteinG sensor chips. However, the default CFCA form factor is stated to only be accurate for CM5-based chips, not including other sensors.

To first assess the degree of inconsistency across sensor types, the reported default active concentrations of NISTmAb were compared across both flow cell pairs on a ProteinG sensor, ProteinA sensor, PrismA sensor and CM5 sensor with ProteinG pre-conjugated. Each CFCA run was performed with NISTmAb at 4 dilutions (50nM, 25nM, 5nM, and 2nM) and at two flow rates (5µL/min and 100µL/min), injecting only on the second (sample) flow cell. NISTmAb is a stable and highly characterized monoclonal antibody reference standard, which made it an ideal choice to compare sensors.[3, 4] The generated data was processed with Biacore T200 Evaluation Software to perform default calibration-free concentration analysis fitting with reference subtraction. Using MALDI-TOF, the molecular weight of glycosylated NISTmAb was found to be approximately 149kDa. Since the NISTmAb hydrodynamic radius is known to be 4.98nm by the certificate of analysis DLS measurement,[4] the Stokes-Einstein equation was employed to define the diffusion coefficient in water (similar viscosity to PBST) at 20°C:

$$D_t = \frac{kT}{6\pi\eta R_h} \tag{S1}$$

where D_t is the diffusion coefficient, k is the Boltzmann constant, T is the temperature in Kelvin (293.15K), η is the viscosity (water: 1.024 mPa*s at 20°C),[5] and R_h is the hydrodynamic radius, so the diffusion coefficient of NISTmAb at 20°C is 4.211E-11 m^2s^{-1}.

As Cytiva mentions, different sensor chips reported different active concentrations for NISTmAb. However, even the CM5 chip with ProteinG conjugated to it, which should in theory be in agreement with the hardcoded form factor for the CM5 chip in the Evaluation Software,[6] did not report an active concentration close to the expected 67.15µM (10.003mg/mL). Interestingly, flow cell pairs on the same sensor chip on the same Biacore T200 system reported different active concentrations for NISTmAb (Figure S1B), using CM5 sensor chips or other sensor chips. Adding to these inconsistencies, the mass transport rate constant k_t (which is calculated from the form factor[6]) has been predicted to fluctuate by up to 15% between Biacore instruments but the influence of other components (e.g. sensor chip type & lot) was not as clear.[7]

While seemingly antithetical to a technique characterized as "calibration-free", a method was therefore developed to re-calibrate the form factors of each Series S sensor chip used in this study. This was deemed necessary, as variability in the experimental form factor would affect the k_t parameter and, by extension, the reported active concentration.[6] Given that NISTmAb is a highly characterized standard with a well-defined concentration, it was treated as a gold standard to adjust the form factor of each flow cell pair per chip, with an assumed 100% activity in its Fc domain. While the immunoreactive fraction of monoclonal antibodies is often considerably

lower, IRF assays are designed to report specifically on the paratope activity.[8, 9] Since NISTmAb was likely affinity purified against its Fc domain during manufacturing (e.g. Protein A chromatography), molecules with an inactive Fc domain were likely discarded.[10] Considering the CFCA binding rate equation under mass transport that was previously defined[11, 12] and showcased below, chip-specific calibrations could be calculated by assuming that the default form factor (G) was the sole source of error skewing the reported NISTmAb active concentration.

$$\frac{dR}{dt} = L_m * (MW * G * L_r * [A_{bulk}] - k_d * R)/(L_m + L_r) \tag{S2}$$

The dR/dt, L_m, MW, L_r, k_d, and R values should remain consistent whether the algorithm was fit with the default (incorrect) form factor or adjusted (corrected) form factor, since the intrinsic properties of the molecular interaction and the raw NISTmAb CFCA data would not change based on how the data was analyzed. With all variables other than the form factor (G) and active concentration (A_{bulk}) being static, setting a "default" and "adjusted" version of the right-hand side of the above equation condenses to:

$$G_{adj} = G_{unadj} * \left[A_{bulk}^{calc}\right]/[A_{bulk}^{CoA}] \tag{S3}$$

where the default form factor, G_{unadj}, was 0.81 (times 10^9 but this power-scaling was not accessible in the software)[6] and the molar concentration of NISTmAb, $[A_{bulk}^{CoA}]$, was determined to be 67.15µM given that the CoA-reported concentration of 10.003 mg/mL took glycan mass into account.[3] NISTmAb CFCA runs were then re-evaluated with the adjusted form factors, demonstrating concentration agreement across flow cells and across sensor types (Figure S1B). However, it is important to note that the accuracy of a CFCA measurement is reliant on the accuracy of the measured molecular weight and diffusion coefficient. Reprocessing the NISTmAb ProteinG FC2-1 data with theoretical errors in the MW or D value markedly changed the reported active concentration of NISTmAb (Figure S1C). Therefore, the most precise methods available to measure MW and D of the analyte should be utilized to avoid propagating errors to the active concentration measurements. Given that SPR is linearly related to mass bound to the chip, relevant PTMs such as glycosylation should be included in these measurements.

2.2 Supporting data for Figure 1

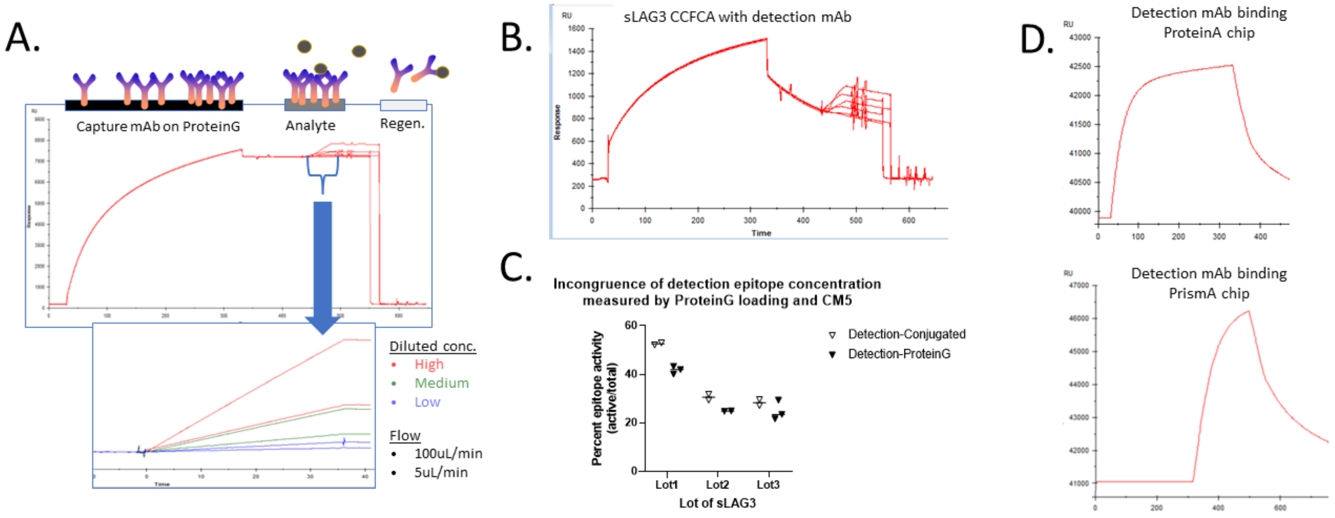

Figure S2: Capture mAb bind ProteinG well, but detection mAb does not. (A) The biotinylated capture mAb loads well (>7000RU) onto a ProteinG sensor chip and remains tightly bound at the end of the loading step. The MTL therefore does not change considerably with little mAb dissociating during the CFCA step. Regeneration of the ProteinG chip with pH 1.5 glycine removes the mAb:biomarker complex from the surface so the next CFCA cycle can be reloaded with fresh mAb. (B) The detection mAb for the sLAG3 LBA does not load on ProteinG well and dissociates relatively quickly from the surface. An in-line CFCA run does report an active concentration for the detection mAb epitope, but (C) the detection mAb active concentration measured by direct amine coupling the sulfo-tagged detection mAb to the chip did not completely agree with this ProteinG loaded CFCA method (three independent replicates of the Detection-ProteinG, two independent replicates of the Detection-Conjugated (CM5)). (D) ProteinA and PrismA chips were also tested to see if the detection mAb would bind with higher affinity to another antibody binding protein, but similar fast off-rates were seen with all three sensor types.

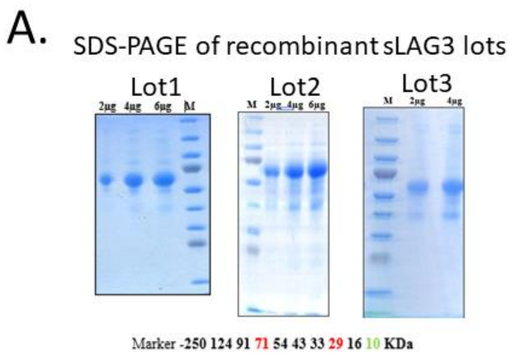

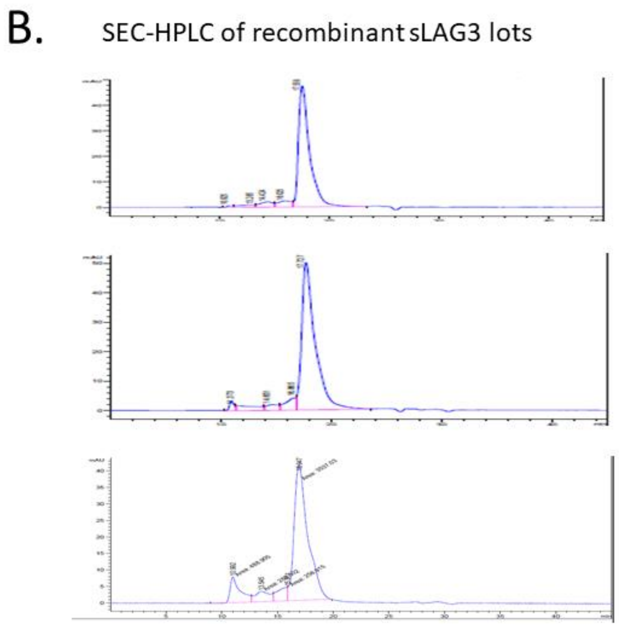

C.

sLAG3 Lot	SDS-PAGE purity	SEC-HPLC purity
Lot1	85.6%	87.2%
Lot2	64.2%	85%
Lot3	69.9%	76.7%

Figure S3: The purity of sLAG3 lots used in this study. (A) SDS-PAGE of 2ug, 4ug, and/or 6ug of the recombinant sLAG3 lot. The main species runs between 54 and 71kDa band in the denatured form. (B) SEC-HPLC of each lot of sLAG3. (C) The purity of the main species was calculated by integrating the main peak vs all other protein staining[13] or A280 signal for SDS-PAGE (4ug lane) and SEC-HPLC respectively.

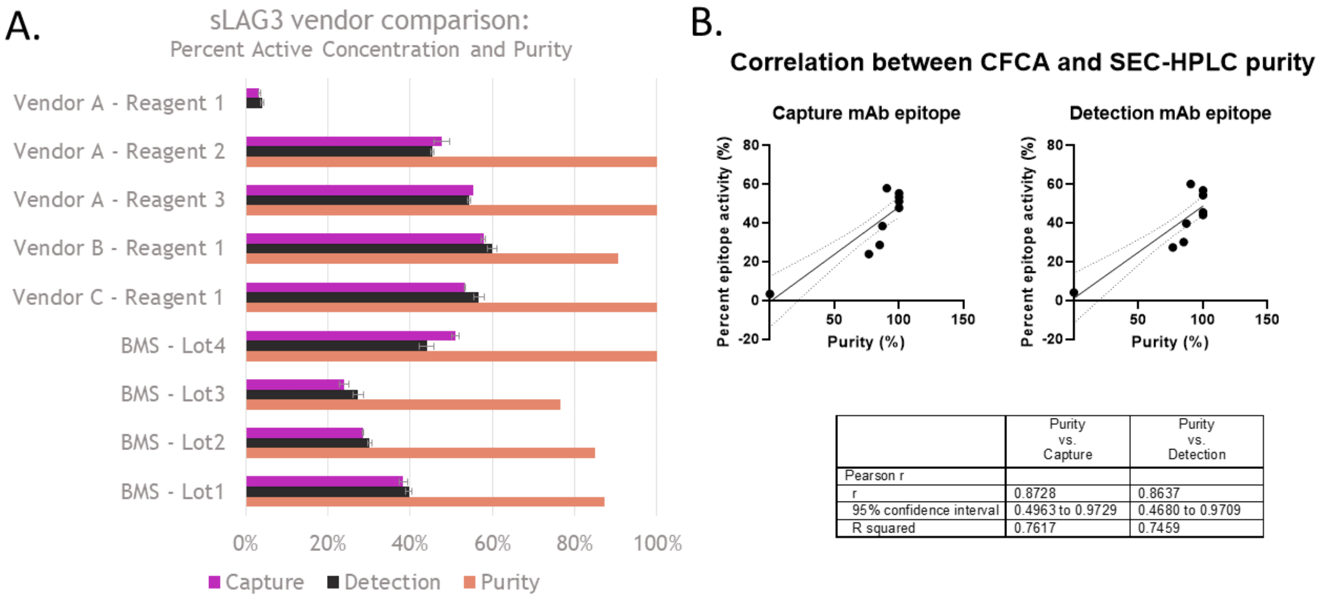

	Purity vs. Capture	Purity vs. Detection
Pearson r		
r	0.8728	0.8637
95% confidence interval	0.4963 to 0.9729	0.4680 to 0.9709
R squared	0.7617	0.7459

Figure S4: Reagent purity moderately correlates with epitope-specific activity, but CFCA activity still varies between reagents with high purity. (A) sLAG3 reagents produced at BMS or purchased from vendor suppliers were assessed for purity, capture epitope active concentration, detection epitope active concentration, and total concentration. The total concentration of each reagent was assessed by BCA following the manufacturer protocol for comparability. Purity was assessed by SEC-HPLC, quantifying the area under the curve for the expected main protein peak (not in the void volume or near the salt peak) compared to the total area under the curve. Capture mAb CFCA was performed by loading on a ProteinA chip while detection mAb CFCA was performed by directly conjugating detection mAb to a CM5 chip. Vendor A – Reagent 1 was expressed in *E. coli* while all other reagents were mammalian-expressed. (B) The CFCA and purity data in Figure S4A were compared using a two-tailed Pearson correlation, additionally fitting the data with a simple linear regression with 95% confidence intervals (dotted lines).

2.3 Supporting data for Figure 2

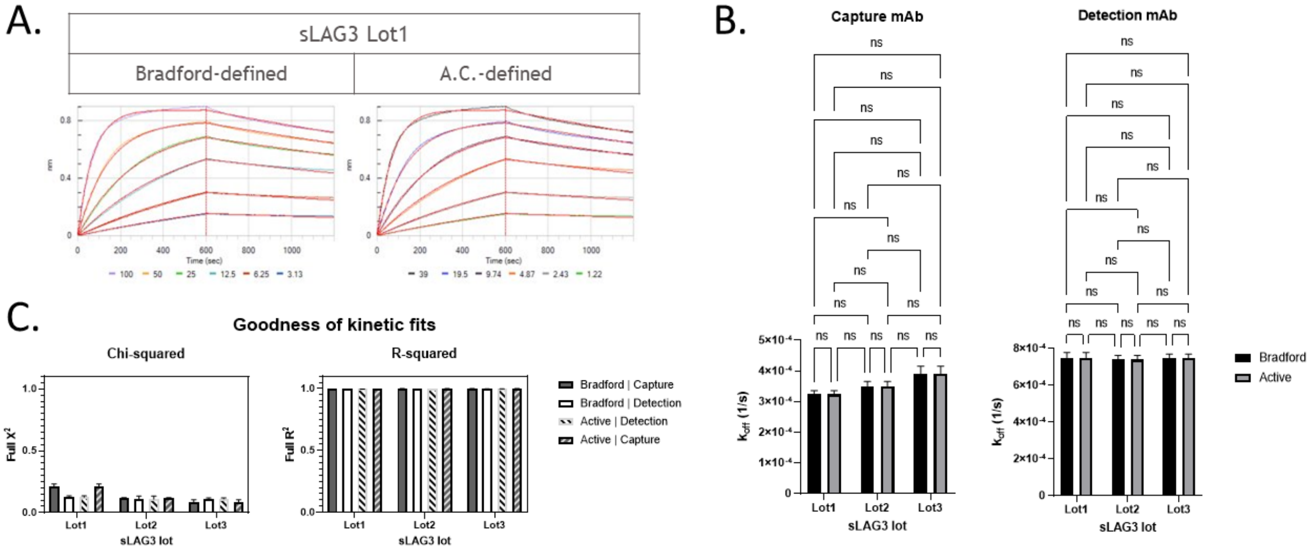

Figure S5: Biolayer interferometry of sLAG3 lots to capture or detection mAbs is well-fit to a 1:1 binding model. (A) A representative binding trace of sLAG3 Lot1 binding to capture mAb that was loaded onto Octet AHC tips. The data was fit either defining the curves by their Bradford or active concentration. The capture epitope active concentration was defined by the average Capture-ProteinG CFCA value, while the detection epitope active concentration was defined by the average Detection(u)-CM5 concentration from Figure 1B,C. (B) Related to Figure 1D&E, the off-rates for each global 1:1 fit of mAb:sLAG3 was highly consistent. No appreciable differences were observed between lots or between using the total or active concentration to define the analyte in the fitting software (n=2). (C) The goodness of fit of each 1:1 kinetic fit of the BLI data is exemplified by the chi-squared and R-squared values. Both metrics demonstrate acceptable goodness of fits, whether defining a calibrator by the Bradford or active concentration.

2.4 Supporting data for Figure 3

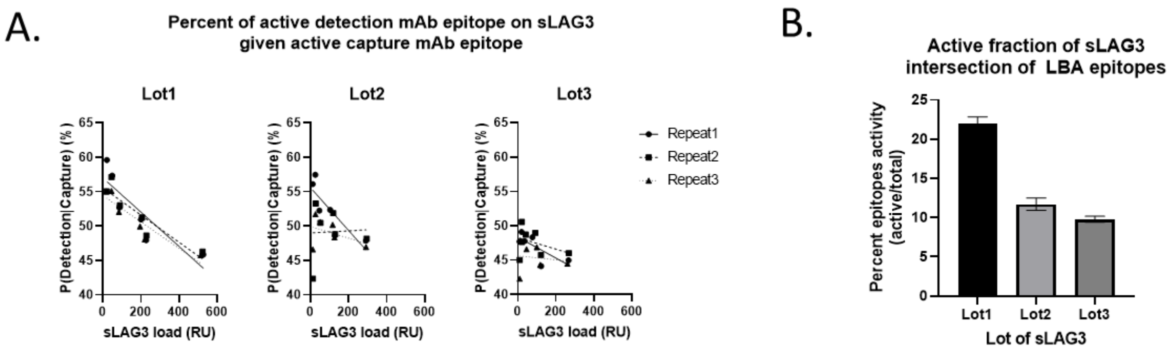

Figure S6: The ideal P(D|C) may be best estimated at low biomarker binding to avoid bivalent interactions. (A) The P(D|C) vs sLAG3 load (RU) of each set of six CFCA injections per lot was analyzed through linear regression to determine the y-intercept (three independent replicates). (B) The percent activity of the intersection, or $P(C \cap D)$, was then calculated by multiplying the respective CFCA-determined percent activity of the capture mAb by the P(D|C).

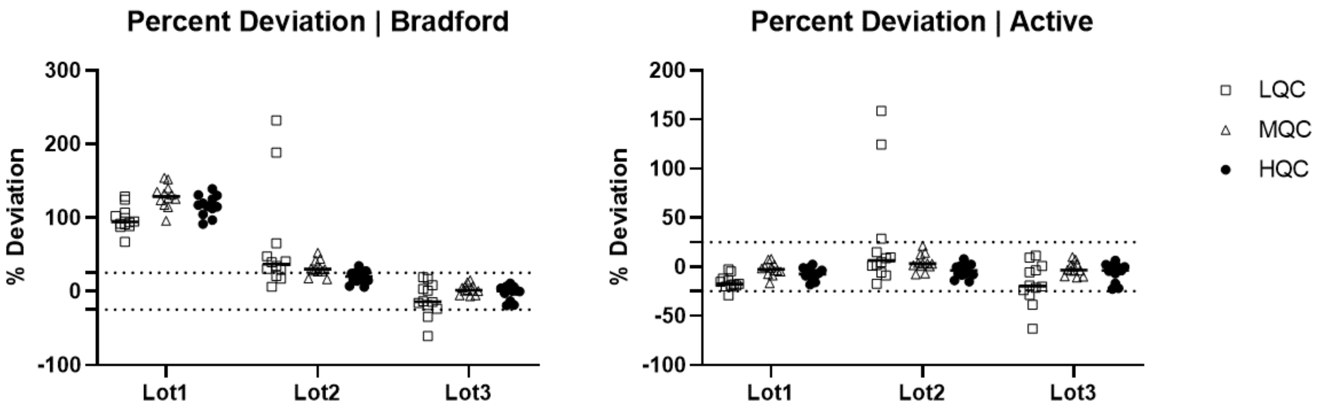

Figure S7: The percent deviation of the back-calculated QC concentrations of each lot compared to a Lot3 standard curve. These plots are related to Figure 3C, but include all data points individually without removing the QCs that failed acceptance criteria to show the full dataset. 12 independent replicates of each QC were run (3 operators with two sets of duplicate QCs per plate). The QC dilutions were aliquoted as Bradford-based concentrations and in post-processing, each QC and the standard curve was transformed to be redefined by their intersection active concentration.

Table S1: Quantification of the variance and deviation of sLAG3 QCs of each lot vs the Lot3 standard curve. Related to Figure 3C and Figure S7, this data demonstrates the nominal concentration of each QC that is prepped, the back-calculated concentration based on the Lot3 standard curve interpolation, the mean %CV of each QC in each set, and the mean %deviation of each back-calculated concentration from the nominal concentration. These values were determined for both the Bradford-based total concentration of each lot and the intersection active concentration of each lot. All QC pairs that passed the acceptance criteria (<25% %CV between pairs) were included in this analysis. Coefficients of variation did not change between the Bradford and active concentrations because both datasets are using the same MSD dataset. So, the differences in %Deviation are likely due to increased relative accuracy of the calibrator concentration.

Lot1						
		Nominal Conc. (ng/mL)	Mean Back-Calc Conc. (ng/mL)	Mean %CV	Mean %Dev	n
Bradford	HQC	50.00	108.07	6.8	116.1	11
	MQC	5.00	11.45	7.0	129.1	12
	LQC	1.000	1.98	8.4	97.9	12
Active	HQC	10.97	10.15	6.8	-7.5	11
	MQC	1.1	1.07	7.0	-2.5	12
	LQC	0.22	0.18	8.4	-15.9	12

Lot2						
		Nominal Conc. (ng/mL)	Mean Back-Calc Conc. (ng/mL)	Mean %CV	Mean %Dev	n
Bradford	HQC	50.00	59.89	7.3	19.8	12
	MQC	5.00	6.57	7.9	31.4	12
	LQC	1.000	1.36	10.6	36.2	9
Active	HQC	5.85	5.62	7.6	-3.9	12
	MQC	0.59	0.61	7.9	4.2	12
	LQC	0.12	0.13	10.6	6.1	9

Lot3						
		Nominal Conc. (ng/mL)	Mean Back-Calc Conc. (ng/mL)	Mean %CV	Mean %Dev	n
Bradford	HQC	50.00	49.12	9.7	-1.8	12
	MQC	5.00	5.14	6.8	2.7	12
	LQC	1.000	0.93	18.6	-6.6	11
Active	HQC	4.9	4.61	9.7	-5.9	12
	MQC	0.49	0.48	6.8	-2.0	12
	LQC	0.1	0.09	18.5	-12.7	11

2.5 Supporting data for Supplemental Discussion

A.

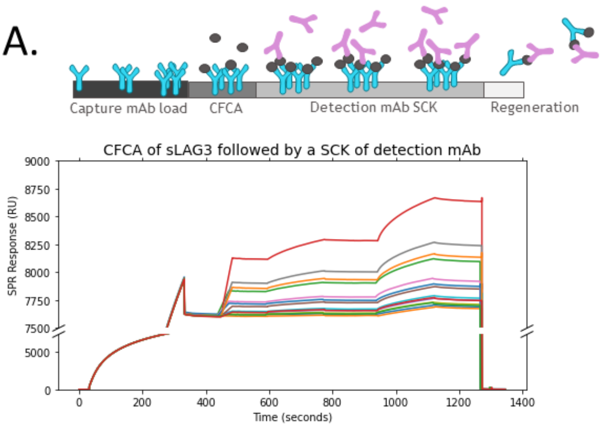

B.

$$\begin{matrix} & km & & ka1 & & ka2 & \\ A_{bulk} & \rightleftharpoons & A + 2L & \rightleftharpoons & LA + L & \rightleftharpoons & LLA \\ & km & & kd1 & & kd2 & \end{matrix}$$

C.

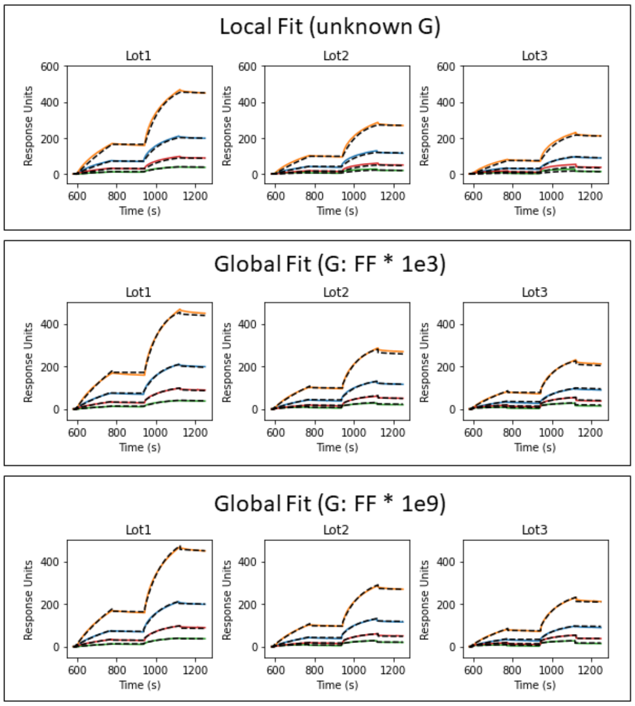

<u>Figure S8: The P(D|C) value can be calculated by nonlinear least squares fitting of the concatenated CFCA and detection mAb injection cycles, but overfitting is a concern.</u> (A) The experimental design for NLLS fitting the P(D|C) is similar to that used in Figure 3A, the capture mAb loading and sLAG3 CFCA experiments were identical. But instead of a single detection mAb injection at 50ug/mL, this strategy was to do a two-concentration single-cycle kinetics experiment using 10ug/mL and 50ug/mL detection mAb. (B) A bivalent analyte model with mass transport was used to fit the reference subtracted and zeroed SCK data. A_{bulk} is the concentration of detection mAb flowed into the instrument, A is the concentration of detection mAb at the sensor surface at any given timepoint, L is the sLAG3 concentration on the surface that is not bound to any detection mAb, LA is the concentration of detection mAb bound to one sLAG3 molecule, LLA is the concentration of detection mAb bound to two sLAG3 molecules, km is the diffusion rate constant, ka1 and ka2 are the association rate constants for the first and second binding event, and kd1 and kd2 are the respective dissociation rate constants. (C) This model was used to fit the SCK data first locally fitting all rate constants and fitting the conversion factor (G) for each lot (top panel). The model was then adjusted so that the on and off-rate constants were fit globally, and the conversion factor (G) could be held constant, but in this globally fit method the km values were still fit locally as each sLAG3 lot could have a slightly different diffusion coefficient and therefore a different km. The middle panel was fit using the chip's form factor (calculated by NISTmAb calibration) * 1e3 since this is closer to the locally fit G value than the 1e9 used in the bottom panel. However, 1e9 is conventionally used to convert molar concentration/rate constant fittings to RU equivalents.[6] All three fits also included bulk shift (RI) fitting locally to each association phase injection.

Table S2: P(D|C) comparison of the Rmax-based method to multiple variations of a bivalent analyte nonlinear least squares curve fit to SCK data. The Rmax-based P(D|C) values were calculated in the main text of the paper, with one concern being that the detection mAb binding injection may not have reached a steady state to be considered an Rmax value. Fitting curves would allow us to instead determine the P(D|C) value using the full kinetic trace. Locally fitting the rate constants and fitting the conversion factor for each lot did yield P(D|C) values higher than the Rmax-based technique, which agrees with the hypothesis that the detection mAb association had not reached steady state. Likewise, the globally fit rate on and off-rate constants with a conversion factor equal to the form factor times 1e3 calculated P(D|C) values higher than the Rmax-based technique. However, when the globally fit model was employed with a conversion factor equal to the form factor * 1e9,[6] the P(D|C) values were curiously low. Overfitting may be an issue with how many variables are manipulatable by the NLLS engine in this model. However, in both globally fit models, the P(D|C) values were Lot3 < Lot2 < Lot1, like the Rmax-based technique.

	Rmax-based	Local fit (G: unknown)	Global fit (G: FF * 1e3)	Global fit (G: FF * 1e9)
Lot1	55.7%	66.5% (G: 1110)	61.0%	38.7%
Lot2	51.6%	69.1% (G: 3089)	58.9%	38.1%
Lot3	47.5%	65.9% (G: 2005)	53.7%	34.4%

3 SUPPLEMENTAL DISCUSSION

3.1 Alternative methods to determine the intersection active concentration of a calibrator

Protein A/Protein G chips were attractive for this initial study since their robust regeneration conditions and reusability allow for direct calibration of each individual chip flow cell and assessment of the active concentration of multiple antibody epitopes per biomarker. However, initial assessment of CM5 chips indicates that the form factor may not vary considerably between chips in the same lot, so that the NISTmAb-calibrated form factor of one CM5 chip may be applied to another chip conjugated with an antibody of interest. While the sLAG3 detection mAb does not bind Protein G well, direct conjugation of the capture mAb or Biotin CAPture kits may be preferrable for other sandwich immunoassay pairs where both mAbs bind Protein A/G to better isolate biomarker-specific signal during the secondary mAb injection. One requirement being that the regeneration conditions would have to be near complete to avoid detection mAb binding biomarker from a previous CFCA cycle. However, more work is required to quantify the form factor variation between chips to ensure form factor consistency from chip to chip.

If each chip must be individually calibrated, there may be cases where the capture mAb cannot saturate a ProteinG/ProteinA surface fully to prevent detection mAb:ProteinG/A binding, potentially obscuring the P(D|C) calculation. Therefore, three additional strategies are outlined for determining the intersection active concentration. (1) Use a ProteinG, ProteinA, or similar antibody-binding standard at a well-defined concentration to calibrate the CM5 chip with the capture mAb already conjugated to the second flow cell (reverse of the NISTmAb calibration) to directly calibrate a CM5 chip. (2) A detection scFv/Fab could be employed to avoid any ProteinA/G binding using the same protocol as described above. (3) A CFCA (without a second mAb injection) can be performed loading capture mAb alone, detection mAb alone, or a 1:1 mixture of capture and detection mAb onto the ProteinA/G chip. This mixture may report on the union of the capture and detection epitopes using the default CFCA algorithm if the MTL was near saturation so that the rate of binding of a biomarker that has both epitopes would be equivalent to the rate of binding of a biomarker with only one of the two. Accurate quantitation of the individual epitope active concentrations and the union active concentration could then derive the intersection active concentration.

$$P(C \cap D) = P(C) + P(D) - P(C \cup D) \qquad \text{(S4)}$$

However, CFCA does not necessarily reach a saturated MTL in practice.[12] It is not yet clear if a more advanced algorithm could account for the differences in MTL in a mixed antibody loaded on a chip to accurately quantitate the union/intersection of the two active epitopes. Additionally, this method assumes that binding the capture mAb does not alter the detection epitope accessibility, and so may be less applicable to harmonizing calibrator lots for sandwich immunoassays than the former method.

3.2 Areas for method improvement to enhance measurement accuracy

(1) Given the reliance on molecular weight in CFCA calculations, the most accurate technology available should be employed to determine the MW of the recombinant biomarker and secondary mAb. However, highly accurate MW measurements (such as ESI-MS) highlight a potentially flawed assumption in the CFCA algorithm: glycoproteins often have heterogeneous molecular weights, but the CFCA algorithm assumes a single species of known diffusion coefficient and molecular weight. Here, using the average MW of a glycoprotein calibrator appears to produce reliable CFCA results that far outperform total protein concentration methods to unify calibrator lots for a sandwich immunoassay. However, accounting for heterogeneous species may help improve accuracy further.

(2) In this work, the diffusion coefficients were theoretically calculated from the molecular weight instead of experimentally determining both. However, this work demonstrates that errors in the diffusion coefficient value can propagate to errors in the active concentration calculated from a dataset. Since the theoretical methods make assumptions about protein shape and specifically use water instead of the assay conditions, it may be preferrable to use an experimentally determined diffusion coefficient for CFCA if absolute accuracy is desired. But if the goal is instead just to harmonize calibrator lots, a theoretical diffusion coefficient may suffice, as the errors in the assumptions would likely be equivalent across all calibrator lots. Additionally, in considering the heterogeneity of glycoprotein molecular weights, it is difficult to envision an experimental method that would be able to accurately measure the diffusion coefficients of each protein species. Whether experimental or theoretical, it is important to note that including the weight of PTMs such as glycosylation in the molecular weight should be strongly considered when defining the MW and D values for the CFCA run, so that the CFCA run calculates a relatively accurate molar concentration for each calibrator lot.

(3) Instead of fitting the NISTmAb data by the default CFCA method to then estimate a new form factor manually, it may be advantageous to directly fit the form factor of the chip flow cell with a static reference standard active concentration. However, the way in which the present study calibrates the system by modifying a single form factor per flow cell may be an oversimplification for two reasons:

(A) Previous studies monitoring the stability and activity of stored antibodies suggests they are extremely stable; with one showing almost no loss in purity or activity over eight years at -70°C,[14] and another suggesting that diagnostic antibodies may have a functional half-life of more than 10 years if stored at 4°C.[15] However, it is not clear whether the NISTmAb reagent is itself losing activity during extended storage at -80°C or during storage at 4°C after thawing. The present CFCA method assumes 100% NISTmAb activity at the ProteinA/ProteinG interface. Given the importance of this calibrant for the consistency of all subsequent active concentration measurements, further work is needed to assess any loss in activity at the ProteinA/ProteinG interface over time.

(B) Previous work argues that at least some of the variability between CFCA experiments can be caused by differences in the flow cell dimensions, relative location of the detection spot, and variability in the flow rate.[7] These variables impact the instrument response nonlinearly through the equation:

$$k_t = 0.98 * \sqrt[3]{\frac{D^2 * F}{h^2 * w * \left(\alpha - \frac{\beta}{2} + 1.5^{-3}\beta\right)}} * G * MW \tag{S5}$$

where k_t is the Biacore-specific transport coefficient, D is the diffusion coefficient, F is the flow rate, h is the height of the flow cell, w is the width of the flow cell, α is the distance from the flow cell inlet to the center of the detection spot, β is the detection spot length, G is the form factor times 10^9, and MW is the molecular weight of the analyte.[6, 7]

The proposed calibration method effectively is trying to adjust the G value to compensate for experiment-specific fluctuations in system variables, which include but is not necessarily limited to variability in the integrated fluidics chip parameters outlined above. While most of the parameters in the cube-root function would remain constant over the course of the experiment, the flow rate changes between cycles of the CFCA run. Because the flow rate is influencing the k_t value non-linearly, it is unclear if a single form factor that linearly adjusts the fit is sufficient to accurately calibrate the system. Given this caveat, the present work maintained the same flow rates (5uL/min and 100uL/min) for CFCA NISTmAb calibrations and sample measurements.

(4) Similar to #3, instead of using the Rmax values to measure P(D|C), a future version of this method may seek to fit the detection mAb injection curves to increase the accuracy of the P(D|C) value. The assumption of 100% 1:1 detection mAb:sLAG3 binding under the bivalent analyte model is unlikely to be perfect. One potential strategy to overcome this issue would be to fit (e.g. using nonlinear least squares) a numerical solution to a bivalent analyte model to the kinetic traces of detection mAb binding to try to attain a more realistic approximation of the system (Figure S8B). The applied form of this model shown below includes mass transport (defined in the system of differential equations below) and a bulk signal correction (RI).

$$\frac{dA}{dt} = -(2 * ka1 * L * A - kd1 * LA) + km * (A_{bulk} - A) \tag{S6}$$

$$\frac{dL}{dt} = -(2 * ka1 * L * A - kd1 * LA) - (ka2 * LA * L - 2 * kd2 * LLA) \tag{S7}$$

$$\frac{dLA}{dt} = (2 * ka1 * L * A - kd1 * LA) - (ka2 * LA * L - 2 * kd2 * LLA) \tag{S8}$$

$$\frac{dLLA}{dt} = (ka2 * LA * L - 2 * kd2 * LLA) \tag{S9}$$

In the above equations, L is the free ligand concentration (sLAG3 on the sensor surface that can bind detection mAb), A is the analyte concentration (Detection mAb), and km/ka1/ka2/kd1/kd2 are the diffusion, association and dissociation constants of the bivalent analyte system. To solve this system numerically, the initial molar concentration of L (L0) would be required. By re-utilizing the form factor (G) to convert between RU and mass bound to the chip, this parameter can be fit using the equation:

$$L0 = \frac{R_{max}^{Biomarker} * P(D|C)}{G * MW^{Biomarker}} \tag{S10}$$

In this way, the P(D|C) becomes a variable solved by the nonlinear least squares engine. With the molar L0 value being directly fit, the SPR response at any given time can be defined as:

$$R(t) = \big(LA(t) + LLA(t)\big) * (G * MW^{DetMAb}) + RI(t) \tag{S11}$$

In the above equations, R(t) is the RU response at time t, LA(t) and LLA(t) are likewise the molar concentrations of LA and LLA species at time t, G is the form factor of the sensor chip flow cell pair, MW^{DetMAb} is the molecular weight of the detection mAb, and RI(t) is the bulk signal correction (refractive index shift) at time t.

In this example, a single cycle kinetics experiment followed each cycle of the CFCA to provide multiple detection mAb concentrations to fit (Figure S8A). The model was first fit locally by each on- and off-rate, diffusion rate constant, and an unknown (to be fit) conversion factor (Figure S8C, Table S2). The model was then adjusted to globally fit the on- and off-rates, locally fit the diffusion rate constants per lot, and include a constant conversion factor that had been calibrated for each sensor chip using NISTmAb. Multiplying the form factor by the base multiplier used for CFCA analysis (1e9) to convert molar concentrations to RU yielded P(D|C) values lower than the Rmax-derived P(D|C) values, which was not expected. But when this multiplier was lowered to be more in line with the locally fit conversion factors (1e3), the P(D|C) values were again above the Rmax-derived values and Lot3 < Lot2 < Lot1, like the Rmax-derived values.

However, one benefit of CFCA over conventional bridging studies is that the method does not require direct comparison between lots to derive an active concentration and unify lots. Fitting the detection mAb binding curves to a bivalent model locally (i.e. for each lot) risks unjustified lot-specific adjustment of the on- and off-rates, potentially reporting incorrect fraction activities. On the other hand, globally fitting the kinetic parameters to define the on- and off-rates of the mAb:antigen interaction is no longer measuring each lot’s active concentration independently. It is therefore unclear how to apply bivalent analyte model curve fitting to potentially better harmonize calibrator lots while retaining the benefits of the above method (e.g. each measurement is lot-intrinsic). Given this, and the general concerns that the NLLS engine may have been overfitting the data given the large number of adjustable variables, the basic Rmax method was chosen to estimate the P(D|C) values for this initial study.

4 SUPPLEMENTAL REFERENCES

(1) Ascierto, P. A.; Lipson, E. J.; Dummer, R.; Larkin, J.; Long, G. V.; Sanborn, R. E.; Chiarion-Sileni, V.; Dreno, B.; Dalle, S.; Schadendorf, D.; et al. Nivolumab and Relatlimab in Patients With Advanced Melanoma That Had Progressed on Anti-Programmed Death-1/Programmed Death Ligand 1 Therapy: Results From the Phase I/IIa RELATIVITY-020 Trial. *J Clin Oncol* **2023**, *41* (15), 2724-2735. DOI: 10.1200/JCO.22.02072 From NLM Medline.
(2) Visentin, J.; Minder, L.; Lee, J. H.; Taupin, J. L.; Di Primo, C. Calibration free concentration analysis by surface plasmon resonance in a capture mode. *Talanta* **2016**, *148*, 478-485. DOI: 10.1016/j.talanta.2015.11.025 From NLM Medline.
(3) Schiel, J. E.; Turner, A.; Mouchahoir, T.; Yandrofski, K.; Telikepalli, S.; King, J.; DeRose, P.; Ripple, D.; Phinney, K. The NISTmAb Reference Material 8671 value assignment, homogeneity, and stability. *Anal Bioanal Chem* **2018**, *410* (8), 2127-2139. DOI: 10.1007/s00216-017-0800-1.
(4) Turner, A.; Yandrofski, K.; Telikepalli, S.; King, J.; Heckert, A.; Filliben, J.; Ripple, D.; Schiel, J. E. Development of orthogonal NISTmAb size heterogeneity control methods. *Anal Bioanal Chem* **2018**, *410* (8), 2095-2110. DOI: 10.1007/s00216-017-0819-3 From NLM Medline.
(5) Tang, B.; Chong, K.; Massefski, W.; Evans, R. Quantitative Interpretation of Protein Diffusion Coefficients in Mixed Protiated-Deuteriated Aqueous Solvents. *J Phys Chem B* **2022**, *126* (31), 5887-5895. DOI: 10.1021/acs.jpcb.2c03554 From NLM Medline.
(6) Pol, E.; Roos, H.; Markey, F.; Elwinger, F.; Shaw, A.; Karlsson, R. Evaluation of calibration-free concentration analysis provided by Biacore systems. *Anal Biochem* **2016**, *510*, 88-97. DOI: 10.1016/j.ab.2016.07.009 From NLM Medline.
(7) Karlsson, R. Biosensor binding data and its applicability to the determination of active concentration. *Biophys Rev* **2016**, *8* (4), 347-358. DOI: 10.1007/s12551-016-0219-5 From NLM PubMed-not-MEDLINE.
(8) Moreno, I.; Dominguez, M.; Torano, A. A kinetic ELISA to determine the immunoreactive fraction of monoclonal antibodies. *J Immunol Methods* **2020**, *476*, 112689. DOI: 10.1016/j.jim.2019.112689 From NLM Medline.
(9) Rhodes, B. A.; Buckelew, J. M.; Pant, K. D.; Hinkle, G. H. Quality control test for immunoreactivity of radiolabeled antibody. *Biotechniques* **1990**, *8* (1), 70-75. From NLM Medline.
(10) Schiel, J. E.; Turner, A. The NISTmAb Reference Material 8671 lifecycle management and quality plan. *Anal Bioanal Chem* **2018**, *410* (8), 2067-2078. DOI: 10.1007/s00216-017-0844-2 From NLM Medline.
(11) Christensen, L. L. H. Theoretical analysis of protein concentration determination using biosensor technology under conditions of partial mass transport limitation. *Anal Biochem* **1997**, *249* (2), 153-164. DOI: DOI 10.1006/abio.1997.2182.
(12) RichaletSecordel, P. M.; RaufferBruyere, N.; Christensen, L. L. H.; OfenlochHaehnle, B.; Seidel, C.; VanRegenmortel, M. H. V. Concentration measurement of unpurified proteins using biosensor technology under conditions of partial mass transport limitation. *Anal Biochem* **1997**, *249* (2), 165-173. DOI: DOI 10.1006/abio.1997.2183.
(13) Schindelin, J.; Arganda-Carreras, I.; Frise, E.; Kaynig, V.; Longair, M.; Pietzsch, T.; Preibisch, S.; Rueden, C.; Saalfeld, S.; Schmid, B.; et al. Fiji: an open-source platform for biological-image analysis. *Nat Methods* **2012**, *9* (7), 676-682. DOI: 10.1038/Nmeth.2019.
(14) Kukis, D. L.; DeNardo, S. J.; Mills, S. L.; Shen, S.; O'Donnell, R. T.; DeNardo, G. L. Stability of monoclonal antibodies, Lym-1 and ChL6, and 2IT-BAD-Lym-1 immunoconjugate with ultra freezer storage. *Cancer Biother Radiopharm* **1999**, *14* (5), 363-369. DOI: 10.1089/cbr.1999.14.363 From NLM Medline.
(15) Argentieri, M. C.; Pilla, D.; Vanzati, A.; Lonardi, S.; Facchetti, F.; Doglioni, C.; Parravicini, C.; Cattoretti, G. Antibodies are forever: a study using 12-26-year-old expired antibodies. *Histopathology* **2013**, *63* (6), 869-876. DOI: 10.1111/his.12225 From NLM Medline.
